# Virtual Lead Identification of Farnesyltransferase Inhibitors Based on Ligand and Structure-Based Pharmacophore Techniques

**DOI:** 10.3390/ph6060700

**Published:** 2013-05-27

**Authors:** Qosay A. Al-Balas, Haneen A. Amawi, Mohammad A. Hassan, Amjad M. Qandil, Ammar M. Almaaytah, Nizar M. Mhaidat

**Affiliations:** 1 Department of Medicinal Chemistry and Pharmacognosy, Faculty of Pharmacy, Jordan University of Science and Technology, P.O. Box 3030, Irbid 22110, Jordan; E-Mails: haneen_pharma@yahoo.com (H.A.H.); hassan-m@just.edu.jo (M.H.H.); drqandil@just.edu.jo (A.M.Q.); 2 Pharmaceutical Sciences Department, College of Pharmacy, King Saud bin Abdulaziz University for Health Sciences, Riyadh 11426, Saudi Arabia; E-mail: qandila@ksau-hs.edu.sa; 3 Department of Pharmaceutical Technology, Faculty of Pharmacy, Jordan University of Science and Technology, P.O. Box 3030, Irbid 22110, Jordan; E-Mail: amalmaaytah@just.edu.jo; 4 Department of Clinical Pharmacy, Faculty of Pharmacy, Jordan University of Science and Technology, P.O. Box 3030, Irbid 22110, Jordan; E-Mail: nizarm@just.edu.jo

**Keywords:** common feature pharmacophore, structure-based pharmacophore, zinc binding group, database screening, CDOCKER, GOLD

## Abstract

Farnesyltransferase enzyme (FTase) is considered an essential enzyme in the Ras signaling pathway associated with cancer. Thus, designing inhibitors for this enzyme might lead to the discovery of compounds with effective anticancer activity. In an attempt to obtain effective FTase inhibitors, pharmacophore hypotheses were generated using structure-based and ligand-based approaches built in Discovery Studio v3.1. Knowing the presence of the zinc feature is essential for inhibitor’s binding to the active site of FTase enzyme; further customization was applied to include this feature in the generated pharmacophore hypotheses. These pharmacophore hypotheses were thoroughly validated using various procedures such as ROC analysis and ligand pharmacophore mapping. The validated pharmacophore hypotheses were used to screen 3D databases to identify possible hits. Those which were both high ranked and showed sufficient ability to bind the zinc feature in active site, were further refined by applying drug-like criteria such as Lipiniski’s “rule of five” and ADMET filters. Finally, the two candidate compounds (ZINC39323901 and ZINC01034774) were allowed to dock using CDOCKER and GOLD in the active site of FTase enzyme to optimize hit selection.

## 1. Introduction

The established link between human cancers and mutant Ras Proteins has prompted considerable concern to target Ras pathway for cancer remedy. Based on such efforts a group of rationally designed drugs targeting the farnesyltransferase (FTase) enzyme which post-translationally modifies Ras oncoproteins has been discovered [1,2].

Ras proteins require farnesylation step (addition of 15 carbon farnesyl moiety to C-terminal cysteine by a thioether bond) which is essential for their binding to plasma membrane and performing their function in signal transduction. This farnesyl moiety is transferred by FTase enzyme to Ras protein bearing C-terminal amino acid sequence known as CAAX motif (C = Cys, A = an aliphatic amino acid, X = is Met or Ser) in the carboxyl terminus of a group of membrane-bound G-proteins [3,4,5,6].

FTase is heterodimeric zinc containing enzyme of two subunits α and β with molecular weights of 49 and 46 kDa, respectively [7]. The α subunit mainly functions to enhance catalysis whereas the β subunit embraces the active site which is composed of two parts; one for the farnesyl pyrophosphate moiety FPP and the other for accommodating the CAAX motif of Ras proteins. The binding of FPP should precede the binding of CAAX motif as it has been proved that CAAX binding will be enhanced by the presence of FPP inside the active site [8].

FTase is classified as a metalloprotein, in which the metal-binding capabilities are encoded in the primary sequence that further plays a crucial role in determining the three dimensional structure [9,10]. The catalytic zinc is identified in the active site of the β subunit and is considered an integral component of FTase both structurally by forming coordinate bonds with active site residues His 362, Asp297, and Cys299 and catalytically most likely by activation of the cysteine thiol of the protein substrate for nucleophilic attack [11,12,13].

Various research groups have published structure and ligand-based pharmacophores in order to reveal the paramount features in FTase’s active site for inhibitor binding. However, they treated zinc atom as hydrogen bond acceptor and ignored the fact that the main determinant of inhibitors' binding to this enzyme is the presence of zinc cation [14,15,16]. Therefore, in the present study, we have inaugurated a pharmacophore hypothesis using Discovery Studio 3.1 (DS 3.1, 2011) (Accelrys Software Inc, San Diego, CA, USA) software by extracting various inhibitors that were co-crystallized with FTase enzyme in order to obtain a pharmacophore hypothesis that is able to afford rational hypothetical vision of the importance of the zinc cation as the main recipient group of FTase inhibitors. Within this pharmacophore, we added a feature called “zinc binding group” to the other available features within DS 3.1 to selectively treat zinc atom as a distinctive and important feature.

## 2. Experimental Section

### 2.1. Generation of Pharmacophore Hypotheses: Common Feature Based Approach

**Figure 1:**
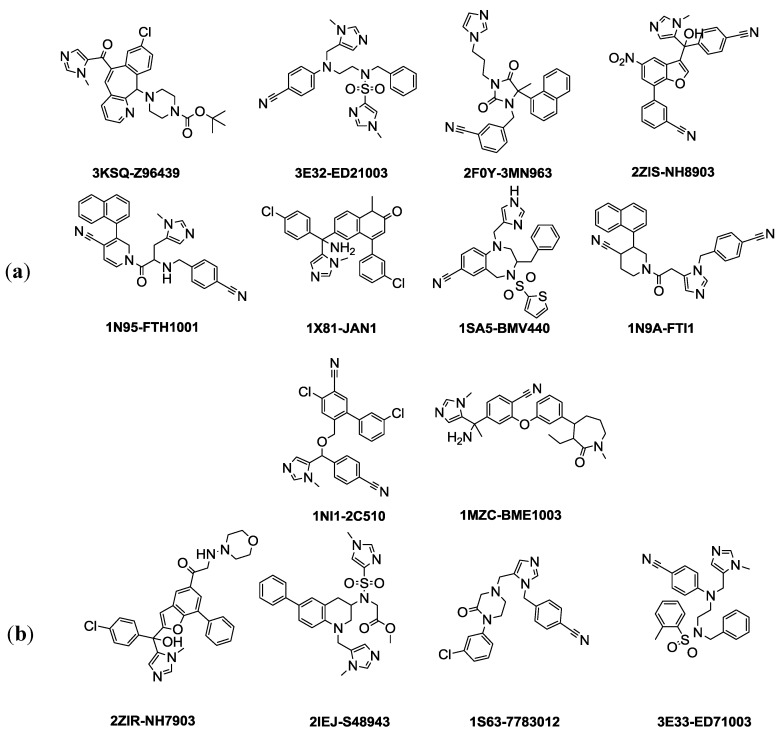
(**a**) Training set ligands utilized in common feature pharmacophore generation are shown with their PDB code and inhibitor name. (**b**) Test set ligands used for common feature pharmacophore validation step.

**Figure 2:**
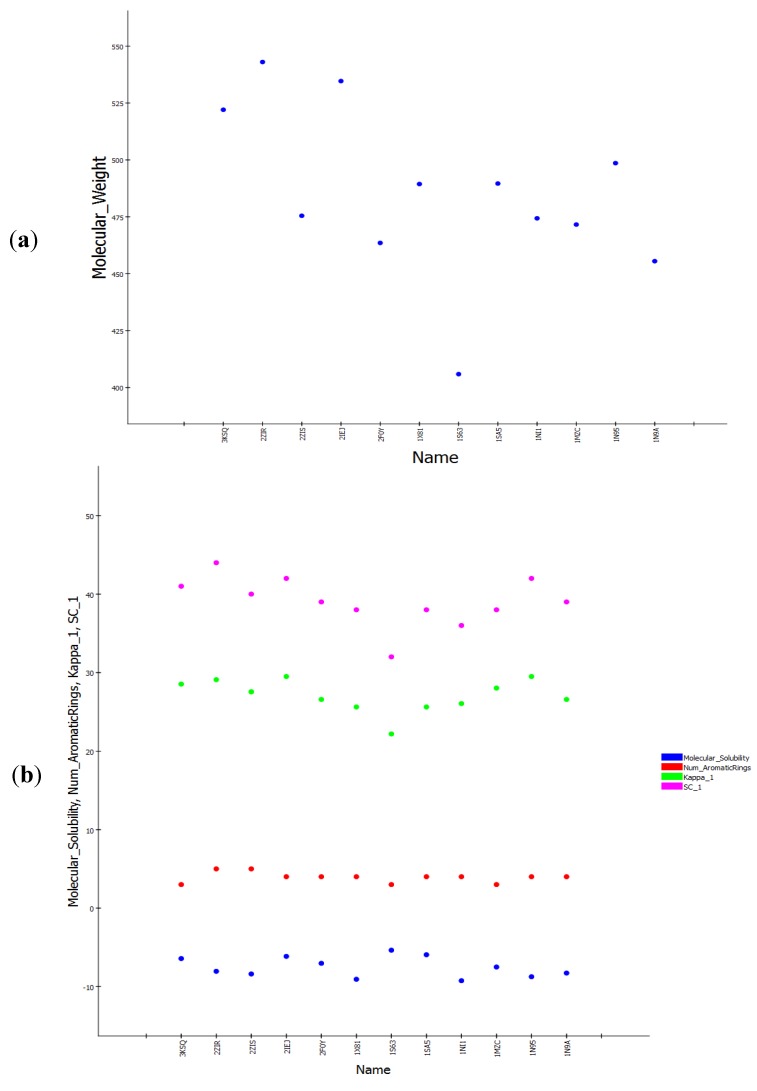
Molecular properties distribution of the fourteen crystal structures used for common feature pharmacophore. (**a**) Molecular weight scale. (**b**) Molecular solubility, Number of aromatic rings, Kappa_1, and SC_1.

Common feature-based pharmacophore modeling is conducted by choosing highly active ligands for a certain target and then extracting the most important functional groups that contribute to activity. Within this pharmacophore hypothesis, twenty three crystal structures of farnesyltransferase with their inhibitors at different resolutions have been collected from the protein data bank (PDB), and then the ligands were extracted from the active sites. Fourteenth co-crystallized inhibitors were chosen as drug-like compounds based on the Lipinski rule of five filter using the Accelrys DS 3.1 *Filters ligands using Lipinski and Veber Rule* protocol. As the extracted ligands are crystallographically determined biological conformations, there is no need to perform *Diverse conformation generation* protocol in order to cover the possible biological space. The fourteen selected compounds were divided into two sets, a training set and a test set by exploiting a protocol in DS 3.1 called *Generate training and test data* where the splitting method is based on structural diversity of the ligands and the splitting percentage for the training set is 70% (Figure 1). The *Common feature pharmacophore generation* protocol was used to generate ten pharmacophore hypotheses, shown in (Table 1), using training set of ten compounds and test set of four compounds as internal validation step (Figure 1). The chemical space of the fourteen compounds was investigated by calculating related molecular properties including chemical and topological properties such as molecular weight, molecular solubility, number of aromatic rings, kappa_1, subgraph count (SC_1) (Figure 2).

**Table 1:** Common feature pharmacophore hypotheses generated based on training set compounds.

Pharmacophore Hypotheses ^a^	Features ^b^	Rank ^c^
1A	**RHAA**	**89.866**
2A	**RHAA**	**89.741**
3A	**ZRHA**	**88.981**
4A	**HHAA**	**88.370**
5A	**HHAA**	**88.132**
6A	**ZRHA**	**87.756**
7A	**RHAA**	**87.643**
8A	**RHAA**	**86.766**
9A	**HHAA**	**86.448**
10A	**RHAA**	**86.374**

^a^ A: related to common feature pharmacophore. ^b^ H, hydrophobic; A, hydrogen bond acceptor; R, ring aromatic; Z, zinc binder. ^c^ The higher the score, the better training set compounds fit to the pharmacophore hypothesis.

In the *Common feature pharmacophore generation* protocol no conformation generation was performed, *Principal* and *MaxOmitFeat* values of 2 and 0 were set for all compounds in the training set as the chosen compounds were the most active and they also were co-crystallized in the active site of the enzyme. Since the active site of the FTase enzyme contains a zinc cation and of all the ten chosen compounds interact with that zinc cation by performing a coordination bond, the features selected to generate the hypotheses were *hydrogen bond acceptor 05*, *hydrogen bond donor 05*, *hydrophobic 05*, *ring aromatics, zinc binder 05*, *maximum pharmacophores* 10, *minimum interfeature distance* 2.0Å. All other control parameters were kept at their default values. The zinc binder feature was modified to be able to identify the zinc binding groups in the active inhibitors that are not included originally in DS 3.1. The best common feature of pharmacophore hypothesis (Pharm-3A) was selected based on the inclusion of the zinc binder after customization in its features (Table 1).

### 2.2. Generation of Pharmacophore Hypotheses: Structure-Based Approach

Structure-based pharmacophore modeling has been extensively implemented by researchers world-wide to provide successful novel drugs with potent activity. Mainly, it is used whenever there is a shortage of information of ligands that bind to the receptor or to get more insight into the geometry of the active site. In this study, a crystal structure of FTase enzyme with a bound ligand (PDB code: 3E33) crystallized at 1.9 Å resolution was utilized to generate the structure-based hypothesis. A sphere of 10 Å radius which covers the most important residues that bind with the twenty three crystallized ligands was generated using *binding site* tools available in DS 3.1. These important residues were assigned by careful studying of the binding nature by which each one the 23 crystallized ligands connect to the active site using the protocol *Receptor-Ligand Pharmacophore Generation*. Pharmacophoric features from the active site were then generated by employing *Interaction Generation* protocol. This protocol is capable of identifying *hydrogen bond donors* (HD), *hydrogen bond acceptors* (HA) and *hydrophobic* pockets (HY) by referring to the active site residues. As a final step to optimize the structure-based pharmacophore, *Edit and Cluster* tool was utilized to cluster and remove any redundant features or features with no catalytic importance.

### 2.3. Validation of the Pharmacophore Hypotheses

Validation was conducted on three separate levels; the first level was performed using the ligand pharmacophore mapping protocol, where the four test compounds mapped to the generated pharmacophores. In the second level a set of 286 ligands extracted from literature and divided into active and inactive molecules based using activity of 500 nM as threshold. Running validation through the mapping method will test the ability of the generated pharmacophores to distinguish the active molecules from inactive molecules. The last step in this level, *Ligand pharmacophore mapping* Protocol available in DS 3.1 with *Best flexible fitting* method was used to measure the extent to which the active molecules could match the pharmacophore and the results were represented by percentages. Finally in the third level, within the common feature pharmacophore , an internal validation performed by providing a set of active and inactive molecules where the results presented as roc curve files.

### 2.4. Database Screening

The generated pharmacophore hypotheses based on the aforementioned two approaches were used as 3D queries to extract chemical compounds from commercially available databases. This process will help in finding potential leads for this target that can be optimized further by investigating drug likeness and synthetic feasibility. A selected compound from the screening method should map all the pharmacophoric features in order to be considered a hit for the common feature pharmacophore hypothesis. On the other hand, a one feature miss was tolerated for considering a compound to pass the screening for structure based pharmacophore. The screening processes were performed using *Ligand Pharmacophore Mapping protocol* with *Best Flexible Conformation Search* method. The candidate molecules were further subjected to numerous filters in DS 3.1 (Lipiniski’s and Veber rules) to select the drug-like compounds. The designated molecules were considered in molecular docking stage.

### 2.5. Molecular Docking

Molecular docking was conducted using *CDOCKER* docking protocol in DS 3.1 and GOLD (Genetic Optimization for Ligand Docking) program version 5.1 from the Cambridge Crystallographic Data Centre (CDCC). CDOCKER uses a CHARMm-based molecular dynamics (MD) scheme to dock ligands within the active site of enzymes and receptors. First, random ligand conformations are generated using high-temperature MD, and then the produced conformations are translated into the binding site. Candidate poses are further created using random rigid-body rotations followed by simulated annealing. A final minimization is used to refine the ligand poses [17,18]. GOLD docking is based on using genetic algorithm to prospect the full flexibility of the ligand with partial flexibility of the active site of the enzyme [19]. GOLD has been validated by over 300 crystal structure complexes extracted from PDB and has showed success in more than 70% of the cases. The crystal structure PDB (3E33, 1.9 Å) [20] of FTase enzyme was selected as it represents the best resolution among all the available crystal structures for FTase in PDB and was prepared using *Prepare Protein* protocol that removes water, corrects any defects in the protein structure and adds hydrogen. The *Input site sphere* was 10 Å in radius using the ligand in the active site as a reference point for both CDOCKER and GOLD.

For CDOCKER, the candidate molecules from the screening process were docked in the active site using CHARMm forcefield with top 10 poses to be presented and scored and keeping the other options in their default values. On the other hand, for GOLD, early termination step was activated if the first three poses have an rmsd value of less than 1.5 Å, other parameters were set as default. The final hit molecules were selected based on a combination of docking score, mode of binding and molecular interaction within the active site.

## 3. Results and Discussion

### 3.1. Common Feature Pharmacophore Hypothesis

The selection of training and test set compounds for pharmacophore hypotheses generation was performed by subjecting twenty three ligands extracted from PDB of FTase enzyme to both Lipinisks’ rule of five; which limits the selection of molecules of appropriate characteristics with respect to size and molecular weight, number of HBD and HBA, and logP values in addition to Vebers’ rules with respect to the number of rotatable bonds and polar surface area, yielding fourteen candidate compounds (Figure 1). In this way selecting the compounds will allow building a pharmacophore hypothesis that possesses the essential properties and at the same time avoids chemical compounds that could cause generated pharmacophore hypothesis to deviate from the optimum configuration. The selected fourteen compounds were further divided into training set and test set using the *Generate training and test data* protocol at DS 3.1. Splitting of these molecules is based on diversity and it is assumed that 70% of them (10 compounds) are the training set. The crystallized biologically active conformations were mapped without any conformation generation as they were considered to possess the required and optimum conformation for pharmacophore hypothesis generation. In this study, the selected features are *HBA*, *HBD*, *hydrophobic*, *ring aromatic*, and *zinc binder*. Ten pharmacophore hypotheses based on common features were generated and they are displayed in (Table 1). According to the table, all the training set compounds fit all the features of the pharmacophore hypotheses and the rank scores ranged from 89.866 to 86.374. Only two of the 10 pharmacophore hypotheses contain Zn^2+^ feature and they are ranked in the third and the sixth positions.

**Figure 3:**
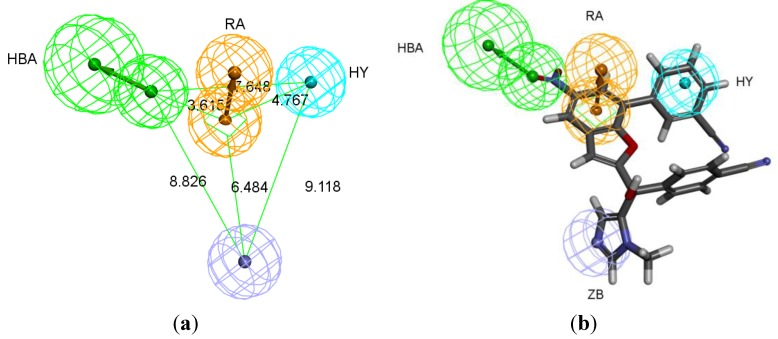
Pharm-3A and its overlay with a training set compound (**a**) Chemical features of pharm-3A with its inter-feature distances. (**b**) 2ZIS-NH8903 overlaid on pharm-3A hypothesis. HY; hydrophobic, RA; ring aromatic, HBA; hydrogen bond acceptor, ZB; zinc binder.

The best pharmacophore hypothesis was chosen based on the fact that the presence of the zinc binding feature is essential. Hypothesis three has showed high values compared to other hypotheses and contains the zinc binding feature, named as Pharm-3A (Table 2). Pharm-3A comprises one zinc binder, one ring aromatic, one hydrophobic and one HBA feature (Figure 3a). Each of the training set molecules has mapped all the features in the pharmacophore hypothesis. For instance, compound 2ZIS-NH8903 has the imidazole ring mapped to the Zn^+2^ binding feature, whereas the nitro group matched the HBA, in addition, one of the aromatic rings was mapped to the ring aromatic feature while the other one was mapped to the hydrophobic feature (Figure 3b).

**Table 2:** Best-fit values of the training and test set compounds based on common feature pharmacophore technique.

PDB ID	Inhibitor Name	Fit Value
		Pharm-3A
2ZIS	NH8903	4.000
1N95	FTH1001	3.643
3E32	ED21003	3.191
1NI1	2C510	3.178
3KSQ	Z96439	3.018
2F0Y	3MN963	2.564
1SA5	BMV440	2.112
1X81	JAN1	1.826
1MZC	BME1003	1.164
1N9A	FTI1	0.788
2ZIR	NH7903 *	3.585
3E33	ED71003 *	3.096
2IEJ	S48943 *	2.748
1S63	7783012 *	1.784

* Compounds used for generating the test set for common feature pharmacophore.

### 3.2. Structure-Based Pharmacophore Hypothesis

This pharmacophore was generated in three stages; the first, *Receptor-ligand pharmacophore generation* protocol was performed for each co-crystallized ligand inside the active site in order to discover the potentially important amino acids that are of strategic contribution to ligand binding. These amino acids will be later considered in the final pharmacophore of the active site. The results collected from running *Receptor-ligand pharmacophore generation* protocol have showed a series of amino acids that contribute to ligand bindings in the active site. Leu96, Tyr93 and Trp 106 performed hydrophobic interaction with lipophilic groups found in the inhibitors’ structures. Tyr361 and Tyr166 have participated in ligand binding by forming ring aromatic interaction with aromatic rings in the inhibitors’ structures. Moreover, Arg202 and Tyr93 were prominent amino acids by being HBDs to their corresponding acceptors in the inhibitors. All the tested inhibitors have showed covalent interaction between the zinc binding group of the inhibitors and the Zn^2+^ inside the active site.

**Figure 4:**
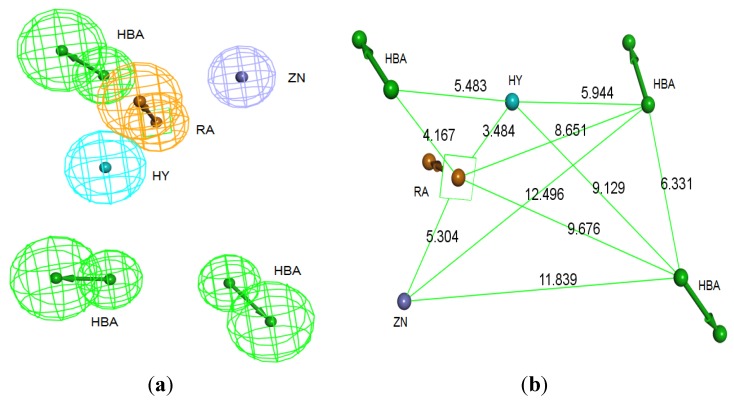
Structure-based pharmacophore hypothesis, Pharm-B. (**a**) Arrangement of pharmacophoric features of Pharm-B. HY; hydrophobic, RA; ring aromatic, HBA; hydrogen bond acceptor, ZB; zinc binder. (**b**) Pharmacophoric features are displayed with inter-feature distances. Tolerance spheres were removed for simplification purposes.

**Figure 5:**
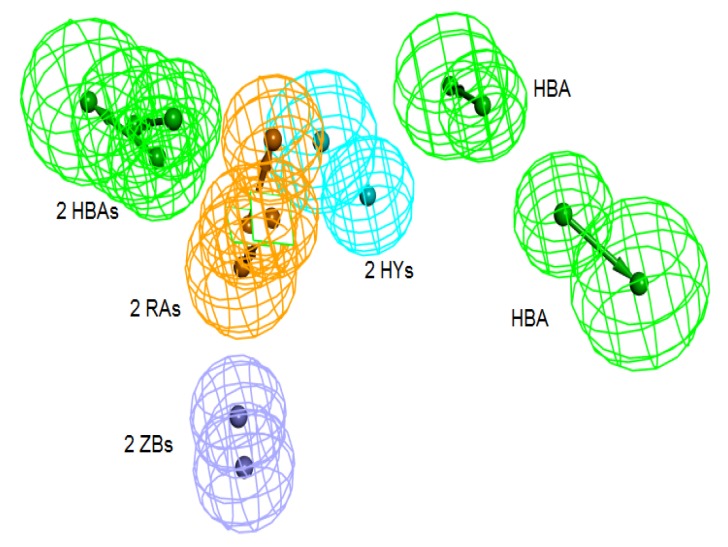
Superimposition of pharm-3A over Pharm-B showing the degree of similarity between the two pharmacophores of RMSD value of 2.52. HY; hydrophobic, RA; ring aromatic, HBA; hydrogen bond acceptor, ZB; zinc binder.

Secondly, *Interaction Generation* protocol was generated based on the lipophilic and the hydrophilic regions within the active site, which was later subjected to further cleaning yielding a pharmacophore hypothesis (Pharm-B) containing six features that are complementary to Tyr361, Tyr166, Phe360, Leu96, Tyr93 and the Zn^2+^ atom. The third stage involved customization of the pharmacophore by selectively replacing HBA feature pointed to the Zn^2+^ atom by zinc feature available on DS 3.1 (Figure 4). The generated pharmacophore from this step is then compared with pharm-3A by utilizing *Pharmacophore comparison* pharmacophore. The results indicated that there was a comparable similarity between the two pharmacophores with RMSD value of 2.52 (Figure 5).

**Figure 6:**
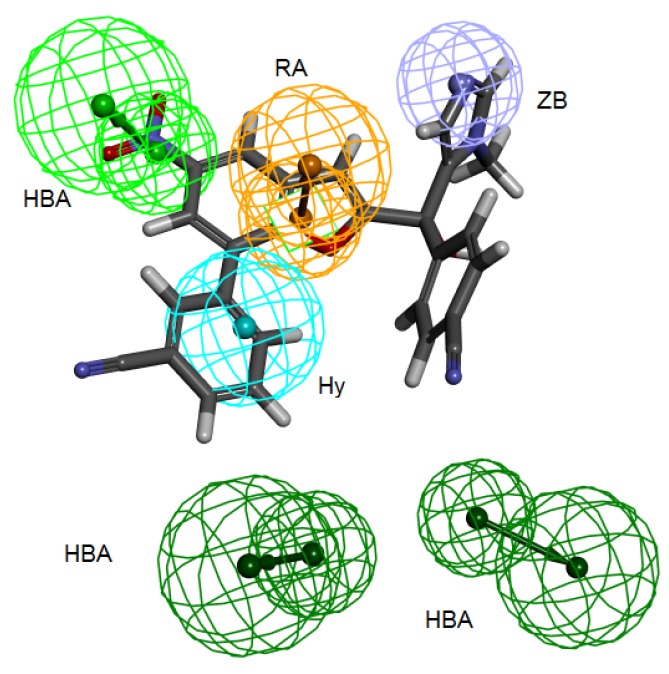
2ZIS-NH8903 overlaid on pharm-B hypothesis. HY; hydrophobic, RA; ring aromatic, HBA; hydrogen bond acceptor, ZB zinc binder.

The fourteen compounds used to generate the common feature pharmacophore Pharm-3A were used to check whether Pharm-B can accommodate these compounds and map them. The fit values were extracted for these compounds which showed good fitting values (Table 3) and all the fourteen compounds mapped at least four features of the six pharmacophoric features of the structure-based hypothesis and every time the zinc binding feature was occupied with zinc binding group. Compound 2ZIS-NH8903 mapping on Pharm-B is depicted in (Figure 6).

**Table 3:** Best-fit values of the training and test set compounds based on structure based pharmacophore techniques.

PDB ID	Inhibitor Name	Fit Value
		Pharm-B
2ZIS	NH8903	5.088
1N95	FTH1001	4.979
3E32	ED21003	5.666
1NI1	2C510	4.713
3KSQ	Z96439	4.324
2F0Y	3MN963	5.457
1SA5	BMV440	4.956
1X81	JAN1	4.627
1MZC	BME1003	4.778
1N9A	FTI1	4.962
2ZIR	NH7903 *	4.933
3E33	ED71003 *	5.266
2IEJ	S48943 *	5.579
1S63	7783012 *	4.167

* Compounds used for generating the test set for common feature pharmacophore.

### 3.3. Validation of Pharmacophore Hypotheses

*Ligand Pharmacophore Mapping* Protocol was used to perform this step. The four test compounds were used to score their fitting to the generated pharmacophore taking into consideration that there is no conformation generation accompanied with rigid fitting technique. The four compounds were found to completely match Pharm-3A with fitting values ranging from 3.58–1.78 (Table 2).

**Figure 7:**
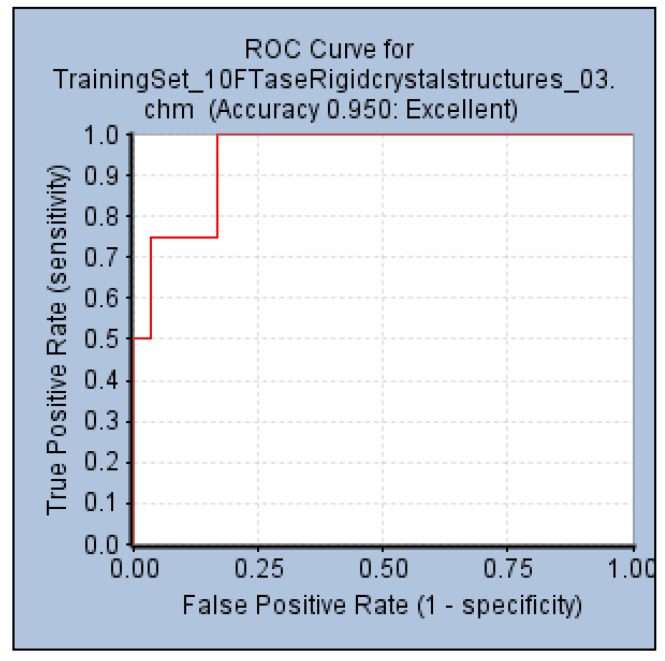
ROC curve of hypothesis Pharm-3A.

An internal validation step performed by generating a ROC curve was also used to confirm the ability of Pharm-3A to be able to distinguish between active and inactive molecules. Within the *Common Feature Pharmacophore Generation* protocol, a validation step was used by providing the active compounds which were the four test set compounds (Figure 1b), and 85 inactive compounds collected from literature. The AUC, accuracy and specificity of Pharm-3A were shown to be the best among all the pharmacophore hypotheses predicted by the training set compounds by recording 93%, 95% and 55% respectively (Figure 7).

In another validation step, a database of 269 compounds of both active and inactive compounds was collected from the literature and drawn using Chemsketch 12.0 and then converted to .sd format file. Of the 269 compounds, 184 were considered active compounds based on their activity against FTase enzyme of more than 500 nM. Using *Ligand Pharmacophore Mapping* Protocol, the 184 compounds were used to investigate the ability of the generated pharmacophore hypothesis to map ligands that are known inhibitors to this enzyme. Of 184 compounds, 153 compounds were mapped on all the features of the common feature pharmacophore hypothesis with a hit rate of 83.15%. And the same compounds were mapped on the structure-based pharmacophore and here, 151 of 184 compounds were mapped on all the features of structure-based pharmacophore with a hit rate of 82.06%.

### 3.4. Database Screening

**Figure 8:**
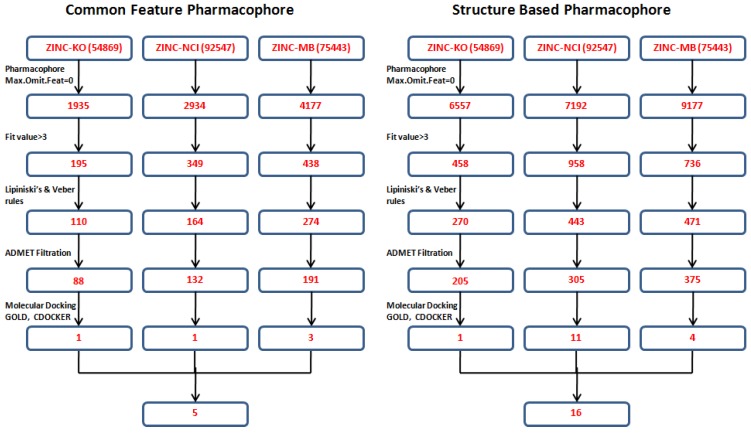
Database screening of three databases employing pharm-3A and pharm-B hypotheses.

Both the common feature pharmacophore and the structure-based pharmacophore hypotheses that were generated previously were subjected to database screening process utilizing *Ligand Pharmacophore Mapping* Protocol using *Best Flexible* search option. These databases are ZINC-NCI_(92547), ZINC-MayBridge_(75443), and ZINC-Key Organics_(54869) [21] which were prepared previously by our group for the screening process. For Pharm-3A, *Ligand Pharmacophore Mapping* protocol was used with *MaxOmitFeat* value of zero as the four features of the Pharm-3A should be fulfilled. A compound will be determined as a hit if it maps all the four features that are included in the pharmacophore hypothesis.On the other hand, a relaxed criteria was used for Pharm-B where the hit is accepted if it can map five features out of the six and hence the *Ligand Pharmacophore Mapping* protocol which allows some of the features to be missed was used. The extracted compounds were then subjected to further screening processes relying on their fitness value, drug likeness according to Lipinski's rule of five and Vebers’ rule. A compound is determined positive according to *Lipiniski* and *Veber* if (i) less than five hydrogen bond donor groups; (ii) less than 10 hydrogen bond acceptor groups; (iii) a molecular weight less than 500; (iv) A LogP value less than 5; (v) number of rotatable bonds less than 10; polar surface area less than 140 Å^2^; and hydrogen bond donors and acceptors less than 12. A subsequent level of filtration to achieve drug like compounds is to perform ADMET screening techniques in DS 3.1 in which the databases are filtered according to their solubility, intestinal absorption and Blood Brain Barrier penetration. A total of 1,296 compounds (885 from the structure-based design and 411 from the common feature pharmacophore) were found to satisfy the assigned filters for the molecular docking step (Figure 8).

### 3.5. Molecular Docking

**Figure 9:**
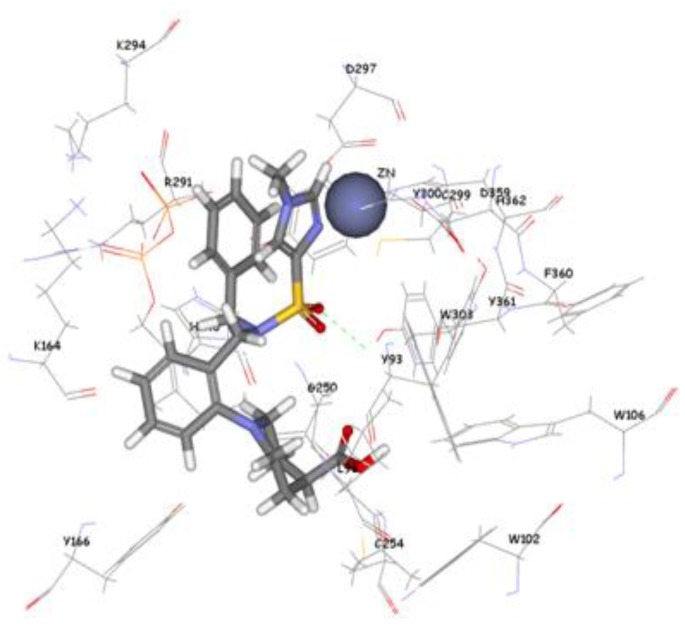
Molecular docking result of ZINC01034774 displayed in stick format, zinc atom CPK and active site amino acids presented as line format.

**Figure 10:**
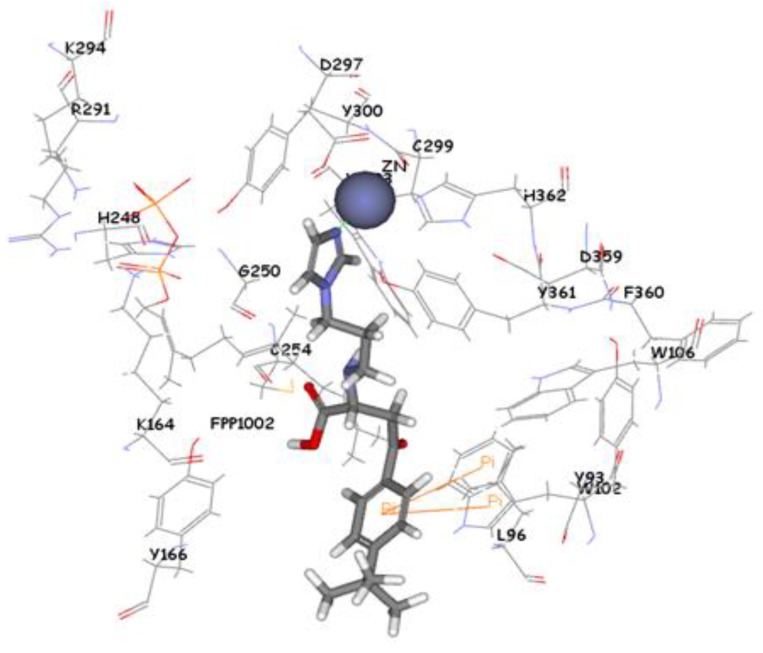
Molecular docking result of ZINC39323901 displayed in stick format, zinc atom CPK and active site amino acids presented as line format.

The 1,296 compounds emerged from both structure and ligand-based pharmacophores conjointly with the training set were submitted to docking programs CDOCKER and GOLD. Consensus scores of the candidate compounds from both CDOCKER and GOLD were performed, eventually compounds enumerated the highest value from score summation of the two docking programs were chosen to be candidate molecules. These scores of the candidate compounds were contrasted by scores obtained for the training set compounds (the highest consensus score for the training set compound is 147.4) and only compounds that scored equally or higher than the training set compounds were fully investigated by studying their binding mode. Of the 1,296 compounds, twenty one compounds had shown scores higher than training set scores. Based on molecular fitting mode inside the active site and their proper interaction with zinc atom, two compounds were chosen as potential virtual leads namely ZINC39323901, and ZINC01034774. Compound ZINC01034774 which was identified from structure-based pharmacophore has a fit value, CDOCKER, and GOLD fitness scores of 3.28, 64.65, and 98.11 respectively. The imidazole ring is supposed to form a coordinate bond with zinc cation which has a perfect pose when the pyridine-like nitrogen points directly at zinc cation of the active site with optimum distance of 1.76 Å. In addition, internal π-π interaction is shown between the imidazole ring and one of the benzene rings of the compound. The other benzene ring is positioned in a hydrophobic pocket within the active site formed from the hydrophobic tail of farnesyl pyrophosphate (FPP), Tyr166, and Lys164. The sulphonamide moiety interacts via hydrogen bond with Tyr361 phenol side chain (Figure 9). Compound ZINC39323901 which was extracted from common feature pharmacophore has a fit value, CDOCKER, and GOLD fitness scores of 3.64, 65.05, and 95.63 respectively. The imidazole ring is in proximity to the zinc cation with a distance of 1.70 Å which is expected to form coordination bond, and the benzene ring is expected to form π-π interactions with indole ring of Trp102 side chain (Figure 10).

## 4. Conclusions

Two distinctive techniques; ligand-based and structure-based drug design techniques were used independently to create two pharmacophore hypotheses, Pharm-3Aand Pharm-B, respectively. The two hypotheses were exploited to find novel inhibitors of FTase enzyme as anticancer agents. Both hypotheses were customized by adding a zinc-binder feature to emphasize the importance of the zinc cation in the active site of this enzyme which is a crucial structural and catalytic feature. Pharm-3A was comprised of four features including a zinc binder, one hydrophobic, one ring aromatic and one hydrogen bond acceptor whereas Pharm-B was composed of six features including zinc binder, one hydrophobic, one ring aromatic and three hydrogen bond acceptors. Both of these pharmacophores were validated for their ability to discover reliable chemical drug candidates. The validation process included internal pharmacophore validation using test set compounds, ROC curve and fit value calculations. All the training and test set compounds have mapped all the features of Pharm-3A and mapped five features of the six in Pharm-B and in both cases; the zinc binder feature was mapped. Three chemical databases were screened and molecules were selected as candidates if they mapped all the Pharm-3A features and at least five features of Pharm-B then using filtering based on Lipinski and Verber drug-like properties and ADMET to acquire favorable drug like properties. Docking of compounds resulted from screening using CDOCKER and GOLD followed by choosing compounds that have higher consensus score compared to the training and test compounds afforded possible candidates which upon studying their binding modes, two potential lead compounds as FTase inhibitors were identified. The structures of all hit compounds will not be revealed at this stage of the research as extensive biological evaluation of these compounds will be under investigation and further steps needed to discover the lead compound.
